# Effects of Biochar and Microbial Organic Fertilizers on Agricultural Productivity and Their Microbial Mechanisms Under Heavy Metal Stress

**DOI:** 10.3390/toxics13110997

**Published:** 2025-11-20

**Authors:** Zhenyu He, Wenming Wang, Bo Kang, Yonggao Yin, Jie Yang

**Affiliations:** 1 School of Civil Engineering, Hefei University of Technology, Hefei 230009, China; hezhenyu707@163.com (Z.H.); yyg2021800035@163.com (Y.Y.);; 2 China Geo-Engineering Corporation, Beijing 100093, China; wangwenming1980@163.com; 3 School of Environmental Science and Engineering, Tianjin University, Tianjin 300072, China; 4 School of Resource and Environmental Engineering, Hefei University of Technology, Hefei 230009, China

**Keywords:** biochar, microbial organic fertilizer, heavy metal adsorption, microbial abundance, heavy metal speciation

## Abstract

Biochar has been demonstrated to be effective in the remediation of heavy metal contamination in soil. However, few studies have examined the impacts of varying proportions of biochar and microbial organic fertilizers on heavy metal adsorption and microbial abundance in soil. Therefore, we investigated the remediation of soil contaminated with heavy metals (Cd and Cu) using different proportions of biochar and microbial organic fertilizer. The results revealed that the adsorption effect of different modifier combinations on heavy metals was notably different, and the metal speciation was significantly altered. Optimal biochar and microbial organic fertilizer combinations significantly reduced the bioavailability and ecological toxicity of heavy metals in the soil, which enhanced plant germination and growth. Furthermore, the addition of modifiers regulated soil pH, preventing root acidification; optimized microbial abundance; enhanced soil microbial environment; and reduced the inhibitory effect of heavy metals on microorganisms. These findings indicate that the addition of amendments may create a virtuous cycle of heavy metal pollutant adsorption, resulting in organic fertilizer efficiency, a better soil environment, and increased crop yield.

## 1. Introduction

Agricultural practices, such as sewage irrigation, fertilizer application, and continuous planting, can cause heavy metal pollution, soil compaction, and other problems. Heavy metal pollution in soil and farmland is prevalent worldwide [1]. In many regions, heavy metals such as Cu, Cd, and Pb considerably exceed the standard limits [2], threatening food security and physical health. According to the Soil Pollution Survey Bulletin, the excess levels of heavy metals in farmland soils in China have reached 16.67% [3]. In China, over 21.95% of the total arable land area is degraded owing to shallow plowing and soil compaction. Several areas are impacted by heavy metal pollution and soil compaction, and hence, both of these issues must be addressed concurrently.

Over the past few decades, strategies such as chemical improvement, biological improvement, and increasing application of organic fertilizers have been used to control heavy metal pollution and soil compaction in farmlands [4,5]. As part of sustainable agriculture development, biochar can be effectively used to increase soil alkalinity, adsorb heavy metal pollution, and provide a favorable environment for the growth and reproduction of soil microorganisms [6,7]. The surface of biochar has a large number of alkali metal ions, such as K^+^, Ca^2 +^ and Na^+^, which may exchange ions with heavy metal ions and adsorb them. Most studies have shown that biochar application decreases the activity and phytoavailability of heavy metals in soil [8,9]. The porosity of biochar can effectively reduce soil compaction, and its addition increases the soil organic matter content and loosens the soil [10,11]. Therefore, optimizing the use of biochar in agriculture to improve long-term crop yield is an urgent issue that needs attention.

The use of organic fertilizer is another important means to improve soil quality, and its effect in improving soil compaction is positive. In recent years, the advent of microbial organic fertilizer has enhanced the effect of organic fertilizer on soil improvement. Microbial organic fertilizer can improve soil compaction while simultaneously regulating the soil flora and improving soil health. Microbial organic fertilizer has been shown to significantly increase the accumulation of dry matter in plant roots, stems, and leaves, increase the number of bacteria and actinomyces, inhibit the growth of fungi, and promote plant growth [12]. However, the sourcing of raw materials for organic fertilizer is complex, and the addition of livestock and poultry manure often causes a high content heavy metal in the organic fertilizer. Long-term use of organic fertilizers on the same land can result in heavy metal pollution risks [13,14]. This is the direction we need to focus on in the future.

Based on the functional characteristics of biochar and organic fertilizer for soil improvement, this study proposes that biochar should be applied simultaneously with microbial organic fertilizer. Therefore, the purpose of our study is as follows: (1) determining the optimal ratio for simultaneous application of biochar and microbial organic fertilizer through the heavy metal removal rate, crop growth, and microbial flora changes; (2) tracking the changes in soil pH value, heavy metal form, and SEM to understand the mechanism of action of the combination of biochar and microbial organic fertilizer to the improvement of soil; and (3) establishing the applicability of the simultaneous application of biochar and microbial organic fertilizer for the improvement of heavy metal-polluted farmland.

## 2. Materials and Methods

### 2.1. Chemicals and Reagents

The China National Pharmaceutical Group Corporation (Beijing, China) supplied the copper sulfate pentahydrate (CuSO_4_·5H_2_O) and cadmium nitrate tetrahydrate (Cd(NO_4_)·4H_2_O) used in this study. Biochar was purchased from Henan Lize Environmental Protection Technology Limited Company (Zhengzhou, China). The maize straw biochar produced by the company underwent pyrolysis at a high temperature; the carbonization temperature was 500 °C; the pH value was 8; the organic carbon content was 410 g/kg; and the total nitrogen and phosphorus contents were 8.51 g/kg and 2.34 g/kg, respectively. Microbial organic fertilizer was purchased from Jiangsu Lvfangyuan Technology Co., Ltd. (Yancheng, China). Biochar and microbial organic fertilizer are commercial products available in the market. All other chemicals were of analytical reagent quality and were used immediately.

### 2.2. Soil Sampling and Basic Analysis

The experiment was conducted in Changfeng County, Hefei City, China (116.87° E, 31.92° N), where a recently acquired agricultural field served as the soil source. The research site experiences a temperate monsoon climate with high humidity, typical of subtropical regions. In the experimental plot, soil sampling was performed using an S-shaped systematic sampling approach, with ten sampling points chosen to ensure spatial representativeness. The collected soil samples were processed by air-drying the topsoil and clay fractions, followed by sieving to remove particles larger than 2 mm in diameter. Subsequently, the physical and chemical properties of the initial soil were characterized.

(1) Soil heavy metal content

**Figure 1:**
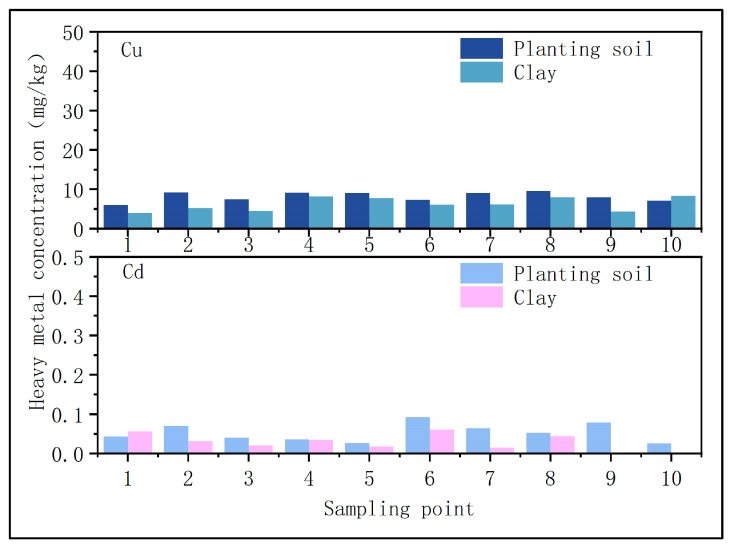
Heavy metal content in surface soil and clay layer of original soil.

The United States Environmental Protection Agency’s Toxicity Characteristic Leaching Procedure (TCLP) is a widely recognized regulatory method for assessing the environmental risk posed by solid waste under challenging leaching conditions. This leaching test is also the most commonly accepted procedure for evaluating the effectiveness of heavy metal remediation in contaminated soils. Field soil samples have been extensively used in toxicity leaching studies to assess contaminant mobility and bioavailability. In this study, the concentrations of heavy metals (including As, Cd, Cr, and Pb) in the test soil were analyzed using inductively coupled plasma mass spectrometry (ICP-MS) after digestion, revealing that the soil exhibited very low heavy metal concentrations, as illustrated in Figure 1.

(2) Soil microbial community structure

DNA extraction, amplification and sequencing: Total genomic DNA was extracted from soil samples. The V3–V4 hypervariable region of the bacterial 16S rRNA gene was amplified with primers 338F (5′-ACTCCTACGGGAGGCAGCAG-3′) and 806R (5′-GGACTACHVGGGTWTCTAAT-3′). Paired-end sequencing (2 × 250 bp) was performed on the Illumina NovaSeq platform by Sangon Biotech Co., Ltd. (Shanghai, China).

Bioinformatic processing: The raw sequencing data were processed as follows. First, primer sequences were removed using Cutadapt (v1.18). Paired-end reads were then merged using PEAR (v0.9.8). Quality filtering was conducted using PRINSEQ (v0.20.4) to trim low-quality bases (quality threshold < 20) and remove short sequences. Operational Taxonomic Units (OTUs) were clustered at a 97% similarity threshold using Usearch (v11.0.667), and chimeric sequences were removed during this process. Taxonomic assignment of representative OTU sequences was performed using the RDP classifier (v2.12) against the RDP 16S rRNA database.

**Figure 2:**
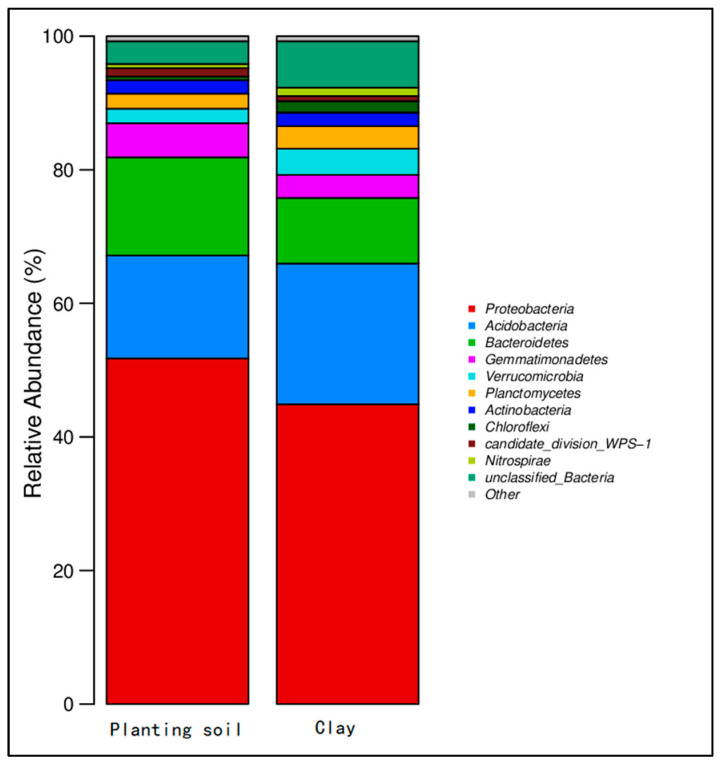
Relative abundance of the top 10 bacterial phyla in planting soil and clay samples.

Statistical analysis: Subsequent analyses of microbial community structure, including alpha and beta diversity, were conducted based on the normalized OTU table. The results of the initial soil microbial community composition are summarized in Figure 2.

(3) Physical and chemical properties of microbial organic fertilizer

To assess the influence of microbial organic fertilizer on the soil during the experiments, the physical and chemical properties of the microbial organic fertilizer were tested before performing the experiment. The results revealed that there were some heavy metals in the microbial organic fertilizer; however, it did not contain the heavy metals chosen for this study (Table 1). The microbial organic fertilizer used in the experiment was confirmed to be free of target heavy metal contaminants, thereby ensuring that the observed changes in soil properties could be attributed to the fertilizer itself rather than external pollution. This provides a reliable baseline for evaluating the impact of microbial organic fertilizer on soil health and heavy metal accumulation.

### 2.3. Experimental Design

All the experimental pots contained 2.5 kg of soil. The surface soil and clay were mixed with biochar, and the clay was placed at the bottom of the pot, and then the surface soil was added. The soil distribution employed in the pot experiment was maintained according to the actual field conditions. In addition, different proportions of biochar (0%, 1%, 3%, 5%, and 7%; soil weight basis) were added, and the experimental groups were designated as BO 0%, BO 1%, BO 3%, BO 5%, and BO 7%, respectively [15]. All five groups received the same amount of microbial organic fertilizer. Neither biochar nor microbial organic fertilizer were added to the control group (CK). The amount of microbial organic fertilizer applied is generally higher than that of traditional chemical fertilizers. Moreover, the organic matter cycle of the soil in the pot experiments was poor. Therefore, to ensure effective planting, the amount of microbial organic fertilizer applied was increased slightly compared with traditional field planting. The amount of microbial organic fertilizer was 10% of the soil weight [16]. Chinese cabbage with a growth cycle of 30 days was used for this experiment, and 20–25 Chinese cabbage seeds were sown in each pot. In order to simulate the growth of plants in the field environment, the test pots were placed in the open air, and the insects were removed manually during the growth process, without rain shelter, shading and other treatments.

The heavy metals, Cu and Cd, were added to the soil to achieve concentrations of 200 mg/kg and 2 mg/kg, respectively. The changes in the heavy metal content and microbial abundance before and after adding biochar were measured, and the filtrate after watering in the pots were collected to measure the content of pollutants. Soil pH and microbial abundance were detected before and after the experiment. The heavy metal content of the planted Chinese cabbages was determined.

## 3. Results

### 3.1. Growth of Potted Plants

**Figure 3:**
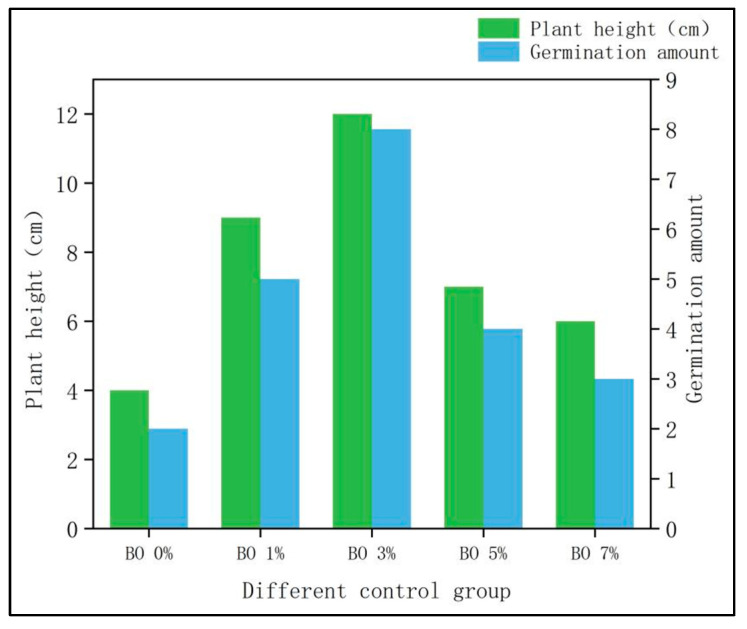
Growth of potted plants.

The plants were counted after examination. The BO 0%, BO 1%, BO 3%, BO 5%, and BO 7% groups bloomed, but the CK group did not. The plant growth is shown in Figure 3.

(1) Plant height: The plant height of Chinese cabbage in the BO 0% group (only microbial organic fertilizer) was 4 cm, while the heights in the biochar-amended groups (BO 1%, BO 3%, BO 5%, and BO 7%) were 9 cm, 12 cm, 7 cm, and 6 cm, respectively. The significant increase in plant height with biochar addition can be attributed to the combined effects of improved soil structure, enhanced nutrient availability, and reduced heavy metal toxicity. Biochar’s porous structure increases soil aeration and water retention, while its alkaline nature helps elevate soil pH, thereby decreasing the bioavailability of heavy metals such as Cd and Cu. Additionally, biochar facilitates the immobilization of heavy metals through adsorption and complexation, reducing their uptake by plants. The optimal plant growth observed in the BO 3% group suggests that this biochar concentration provides the most favorable conditions for root development and nutrient absorption, whereas higher biochar levels (e.g., BO 5% and BO 7%) may lead to nutrient imbalance or limited root space, thus inhibiting growth.
(2) Percentage of germination: The germination rate in the BO 0% group was 9.09%, while the rates in the biochar-amended groups were 21.71%, 34.78%, 16.67%, and 13.04%, respectively. The notable enhancement in germination, particularly in the BO 3% group (34.78%), is likely due to the synergistic effects of biochar and microbial organic fertilizer in mitigating heavy metal stress. Biochar adsorbs heavy metals and reduces their exchangeable fractions, thereby lowering phytotoxicity. Moreover, the addition of biochar improves soil microbial activity and organic matter decomposition, which promotes nutrient release and creates a more favorable microenvironment for seed germination. The decline in germination at higher biochar concentrations (BO 5% and BO 7%) may be associated with excessive adsorption of essential nutrients or the release of inhibitory compounds, such as polycyclic aromatic hydrocarbons, which can adversely affect seed viability and early seedling growth.

To explore the influence of heavy metals on Chinese cabbage grown in each pot, the heavy metal content of the Chinese cabbage was measured. The test results revealed the absence of heavy metals in the Chinese cabbage grown in the experimental pots.

### 3.2. Changes in Heavy Metals in Soil

(1) Migration of heavy metals into soil

**Figure 4:**
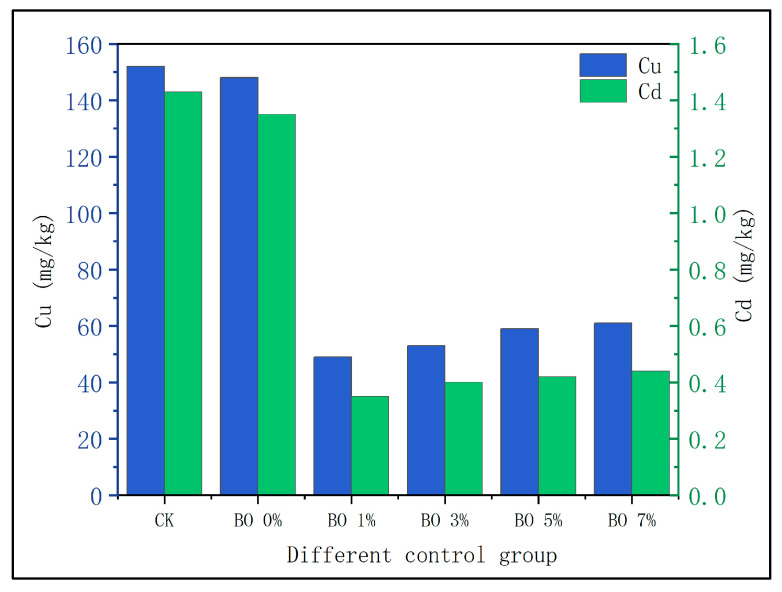
Heavy metal content in potted soil.

The concentrations of heavy metals in the soil and the corresponding filtrates are presented in Figure 4. The heavy metal content in the CK group and the BO 0% group was similar, while the concentrations of Cu and Cd ions in the four biochar-amended groups (BO 1%, BO 3%, BO 5%, and BO 7%) were significantly lower. In the CK group, the removal rates of Cu and Cd were 24% and 26.5%, with residual concentrations of 152 mg/kg and 1.47 mg/kg, respectively. The BO 0% group showed slightly improved removal rates of 26% for Cu and 32.5% for Cd, with residual concentrations of 148 mg/kg and 1.35 mg/kg. In contrast, the biochar-amended groups exhibited markedly higher removal efficiencies: Cu removal rates were 75.5%, 73.2%, 70.3%, and 69.6%, and Cd removal rates were 82.5%, 80%, 79%, and 78% for BO 1%, BO 3%, BO 5%, and BO 7%, respectively. These results are consistent with previous studies reporting maximum adsorption efficiencies of straw biochar for Cd and Cu ions up to 88% and 82.5%, respectively [17,18].

**Figure 5:**
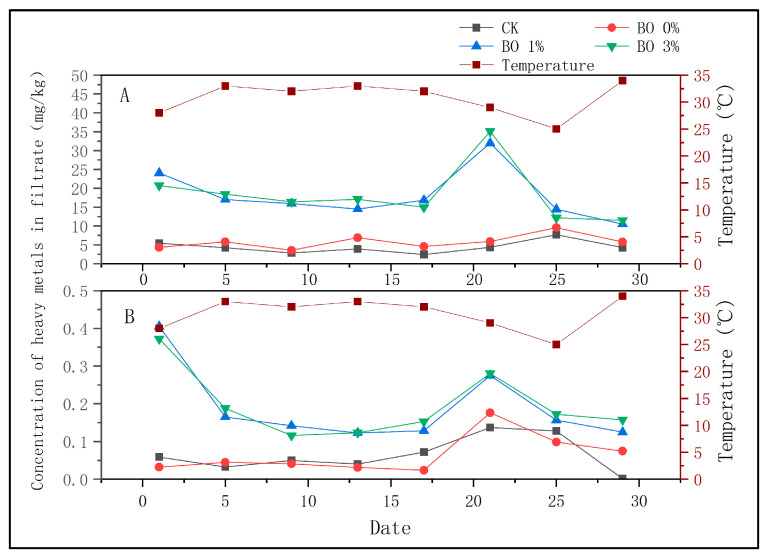
Concentration of heavy metals in filtrate. (**A**) Cupric ion. (**B**). Cadmium ion.

During the experiment, filtrate was collected only from the CK, BO 0%, BO 1%, and BO 3% groups. As shown in Figure 5, the Cu and Cd concentrations in the filtrate generally ranged from 1–10 mg/kg and 0.1–0.5 mg/kg, respectively. Notably, the CK and BO 0% groups exhibited lower filtrate metal concentrations compared to BO 1% and BO 3%. A significant increase in filtrate metal concentrations was observed on the 21st day, particularly in the BO 0% group, where Cu reached 20–30 mg/kg and Cd reached 0.15–0.2 mg/kg. In contrast, the BO 1% and BO 3% groups showed reduced filtrate metal levels, with Cu at 2–15 mg/kg and Cd at 0.05–0.15 mg/kg.

The enhanced metal removal in biochar-amended groups can be attributed to the high specific surface area, porous structure, and abundant surface functional groups of biochar, which facilitate ion exchange, surface complexation, and precipitation with metal ions such as Cd^2+^ and Cu^2+^. The lower filtrate metal concentrations in the CK and BO 0% groups may be due to the lack of a stable adsorption medium, leading to higher metal mobility under leaching conditions. The sudden increase in metal concentration on the 21st day coincided with a rainstorm event, which likely promoted the disintegration of soil aggregates and enhanced metal leaching, especially in treatments without biochar. The presence of biochar mitigated this effect by improving soil structure and water retention, thereby reducing metal mobility and leaching potential.

(2) Morphological changes in soil heavy metals

**Figure 6:**
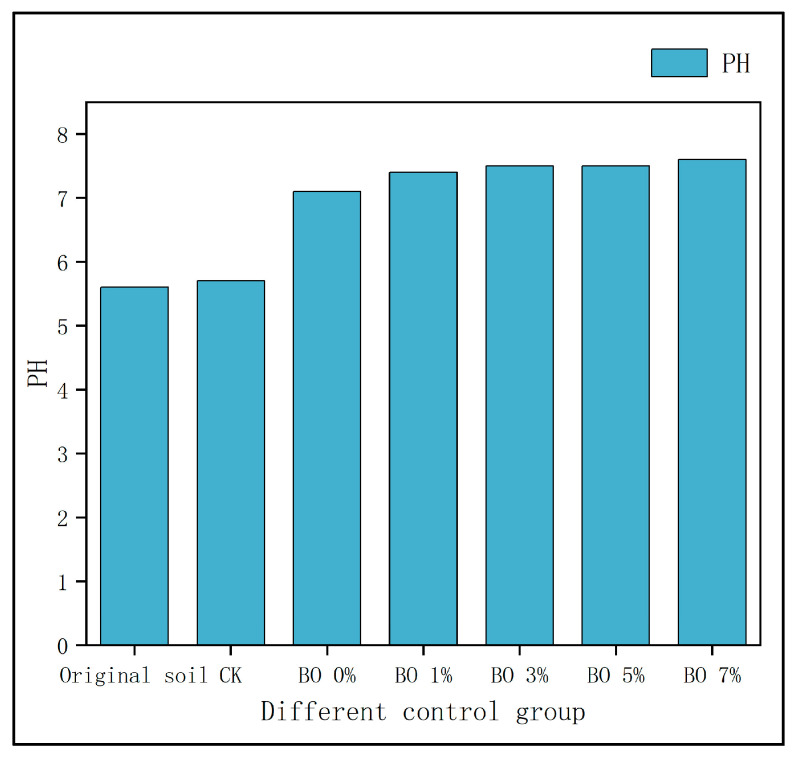
Soil PH value.

The application of biochar significantly influenced soil pH and heavy metal speciation. As shown in Figure 6, the original soil was acidic (pH 5.4), and the CK group maintained this pH. The BO 0% group (with microbial organic fertilizer only) increased the soil pH to 7.2, while the biochar-amended groups (BO 1% to BO 7%) further raised the pH to approximately 7.5, transforming the soil from acidic to weakly alkaline.

**Figure 7:**
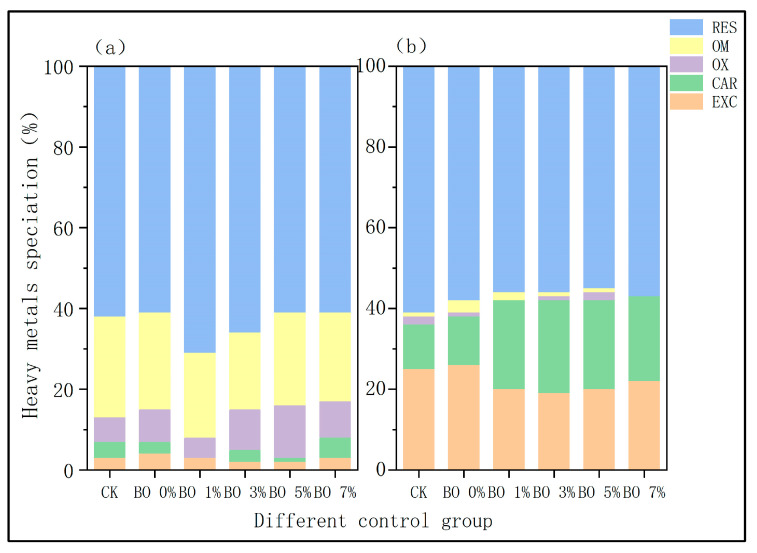
Heavy metal speciation in potted soil. (**a**) Cupric ion. (**b**) Cadmium ion.

Heavy metal speciation analysis (Figure 7) revealed that in biochar-amended soils, Cu was predominantly present in the residual fraction (60–70%), followed by the organically bound fraction (19–23%) and the Fe-Mn oxides-bound fraction (5–15%). The exchangeable and carbonate-bound fractions were minimal. In contrast, the CK and BO 0% groups had a significantly lower organically bound Cu fraction (only ~12%). For Cd, the residual fraction was also dominant in biochar-treated soils (45–60%), with the exchangeable and carbonate-bound fractions accounting for 19–22% and 21–23%, respectively. The CK and BO 0% groups showed higher exchangeable Cd (24–25%) and lower carbonate-bound Cd (~12%) compared to the biochar-amended groups.

The shift in metal speciation toward more stable forms (e.g., residual and organically bound fractions) in biochar-amended soils is closely linked to the increase in soil pH. The alkaline nature of biochar promotes the precipitation and complexation of heavy metals, reducing their bioavailability. The increase in the organically bound fraction of Cu in biochar groups suggests enhanced complexation with organic matter derived from both biochar and microbial organic fertilizer. The reduction in the exchangeable fraction of Cd indicates decreased immediate bioavailability and ecological toxicity. These changes collectively contribute to the immobilization of heavy metals, thereby reducing their phytoavailability and potential environmental risk.

### 3.3. Change in Abundance of Microbial Community in Soil

**Figure 8:**
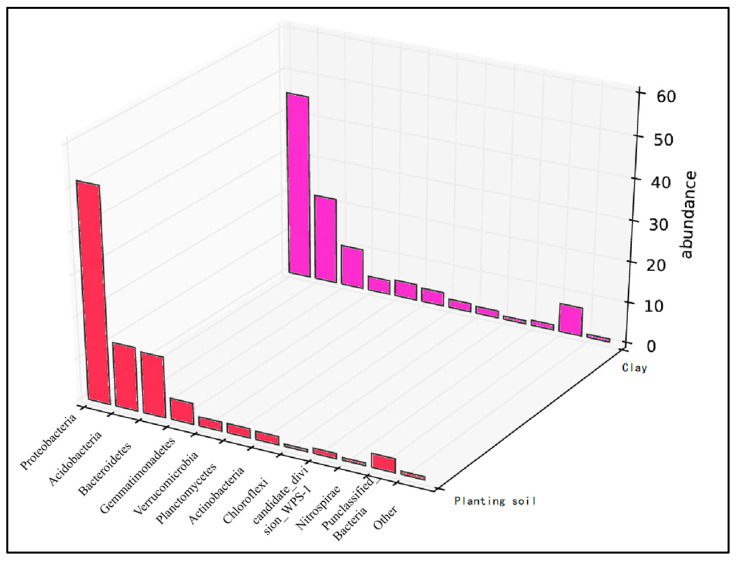
Microbial abundance in soil samples.

**Figure 9:**
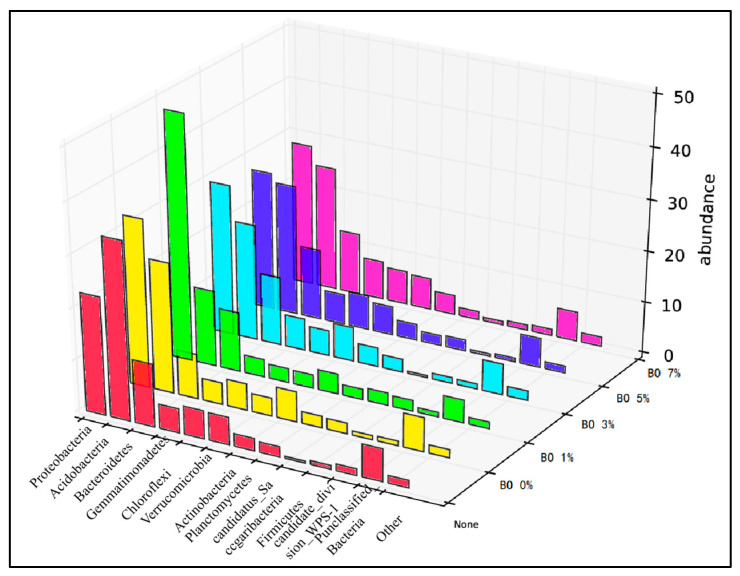
Microbial abundance in soil after the experiment.

Comparing the microbial abundances of cultivated soil and clay in the sampled soil, the soil was mainly distributed in 10 known bacterial phyla and a small number of unclassified groups (Figure 8). The microorganisms detected included Proteobacteria, Bacteroidetes, Acidobacteria, Gemmatimonadetes, Verrucomicrobia, Actinobacteria, Chloroflexi, Candidatus Saccharibacteria, and Planctomycetes. In the planted soil, the abundance of Proteobacteria was 50%, Bacteroidetes and Acidobacteria accounted for 10%. In the clay layer, Proteobacteria accounted for 45%, Acidobacteria accounted for 20%, and Bacteroidetes accounted for 8%. After the experiment, Proteobacteria, Bacteroidetes, and Acidobacteria were dominant (Figure 9). The abundance of Proteobacteria decreased to 25–40%, and the abundance of Acidobacteria increased significantly, accounting for approximately 15–32%.

### 3.4. Soil Microbial and Metabolome Analysis

To investigate the relationship between soil microbial community structure and metabolic function under different amendment treatments, we performed dimensionality reduction and integrative analysis on microbial (16S rRNA sequencing) and metabolomic datasets. Given the distinct nature of these two data types, direct comparison was challenging. Therefore, principal coordinate analysis (PCoA) was first applied separately to reduce the dimensions of both the microbial community data and the metabolomic profile data. Subsequently, Procrustes analysis was utilized to superimpose the PCoA results, allowing for a visual assessment of the overall concordance between the microbial community structure and the metabolic functional profile across different samples.

**Figure 10:**
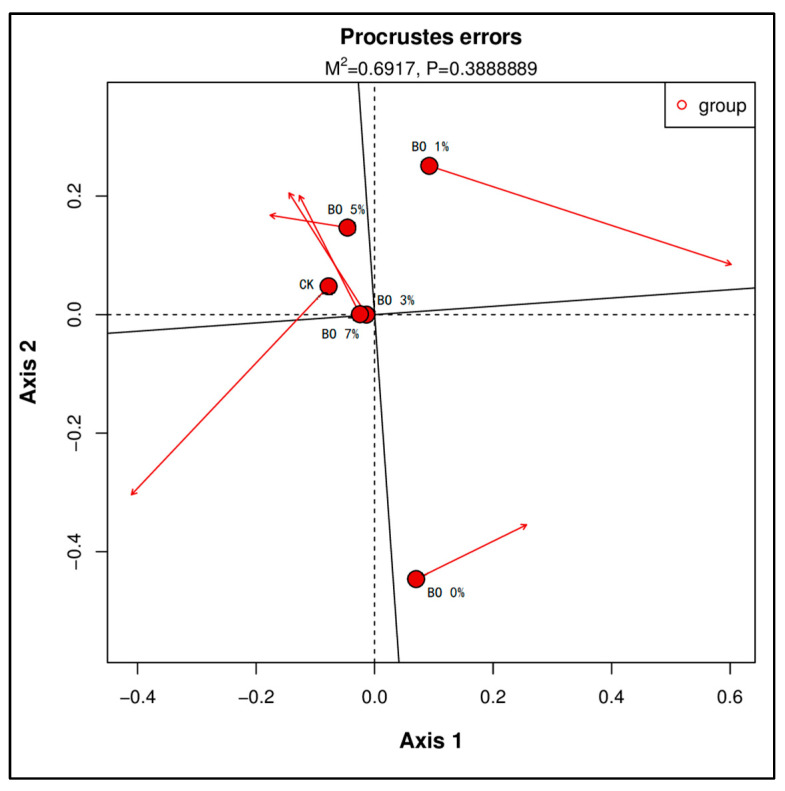
Procrustes analysis. The higher the similarity between samples in the graph, the more clustered the graph. The arrow depicts the offset between species abundance and functional abundance.

The Procrustes analysis result (Figure 10) displays the distribution of samples based on their microbial community structure (points) and their corresponding metabolomic profiles (arrowheads). The length and direction of the arrows connecting each point (microbial community) to its arrowhead (metabolome) represent the magnitude and direction of the discrepancy between the two datasets for that sample. A key observation is that samples from groups with higher biochar amendment levels (BO 3%, BO 5%, and BO 7%) exhibited shorter arrows and more clustered distributions of both points and arrowheads. This indicates a higher degree of similarity or consistency between the microbial community composition and the soil metabolomic profile in these treatments. Conversely, samples from the CK, BO 0%, and BO 1% groups showed longer arrows and more dispersed distributions, suggesting a greater dissociation between the microbial structure and metabolic function under these conditions.

**Figure 11:**
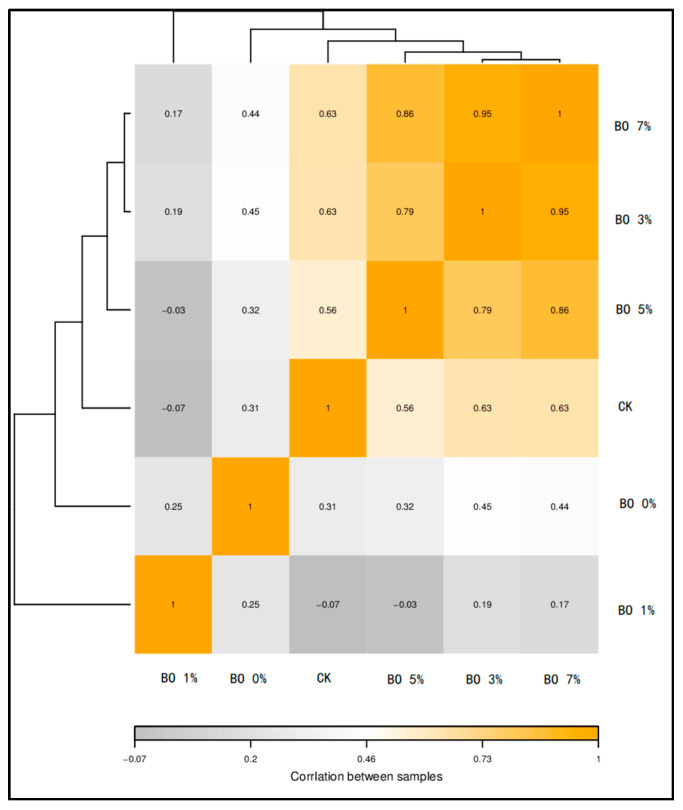
Sample correlation heat map. The color block in the figure represents the correlation index value. The correlation index between the samples decreases as the color becomes grayer. The correlation index increases as the hue becomes yellower.

This finding is further corroborated by the sample correlation heatmap (Figure 11), which illustrates the pairwise correlation coefficients of the overall microbial and metabolomic profiles among different treatment groups. In the heatmap, color blocks represent correlation indices, with yellower hues indicating higher positive correlations and grayer hues indicating lower or negative correlations. The heatmap clearly shows that the BO 0% and BO 1% groups had low or negative correlations with the CK group, indicating distinct microbial and metabolic states. In contrast, the BO 3%, BO 5%, and BO 7% groups demonstrated significantly stronger positive correlations with the CK group and among themselves. This pattern suggests that the addition of biochar, particularly at levels of 3% and above, enhanced the correlation and consistency between the soil microbial community structure and its metabolic functional output. It implies that biochar amendment helped to establish a more stable and coordinated soil micro-ecological system, where shifts in microbial populations were more closely reflected in the soil’s metabolic signature, potentially leading to improved soil functional resilience.

### 3.5. Soil Pore Structure of Different Treatment Groups Under Scanning Electron Microscopy

**Figure 12:**
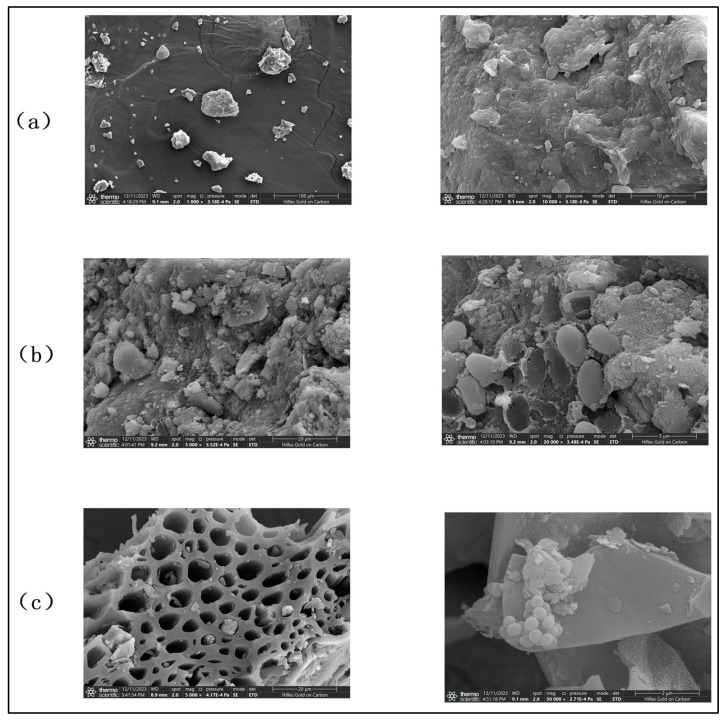
Sample scanning electron microscope: (**a**) CK, (**b**) BO 0%, (**c**) BO3%.

The microorganisms in the soil generally regulate the soil organic matter [19]. The porous microstructure and relatively large specific surface area of the biochar provide a living habitat for microorganisms [20]. Scanning electron microscopy of soil without biochar and microbial organic fertilizer revealed the microstructure of each control group (Figure 12a), which revealed no visible pore structure on the soil surface and no additional microorganisms. In BO 0% (Figure 12b), with only microbial organic fertilizer addition, the surface structure of the soil was more complex than that of the CK group, and a large number of microorganisms were found to be enriched on it. For different control groups supplemented with biochar, BO 3% group with the best plant growth (Figure 12c) has a distinctly porous structure of biochar, and microorganisms can be found in the porous structure of biochar through more detailed observation, which is consistent with the results of previous studies that biochar can provide a living environment for microorganisms [21]. The comparison of soil structure revealed that the experimental group supplemented with biochar and organic fertilizer had higher soil porosity. The scanning electron microscope results of CK group revealed a tendency for soil compaction, and the addition of amendments effectively reduced the harm caused by soil compaction.

## 4. Discussion

### 4.1. Effects of Amendments on Biomass of Chinese Cabbage

The Chinese cabbage in the CK group failed to germinate as a result of the addition of heavy metals. Moreover, in BO 0%, the germination rate of Chinese cabbage was much lower than that of other experimental groups; although, in the heavy metal polluted soil, the addition of microbial organic fertilizer could promote the growth of Chinese cabbage. The Chinese cabbage had varying degrees of germination with the addition of biochar organic fertilizer. The germination and growth of Chinese cabbage increased with the concentration of biochar, reaching its maximum in the BO 3% group. However, in the BO 5% and BO 7% groups, germination and plant height of Chinese cabbage considerably decreased. The results of this experiment are consistent with the results of Buss’ study. With an increase in biochar addition, crop growth first increases and then decreases [22]. The impact of biochar application rates on soil is consistent with other research findings on the use of biochar for treating heavy metal-contaminated soil. Through a three-year field experiment, Wang found that when the single application rate of biochar exceeded 40 t/ha, soil properties showed continuous improvement, and corn yield steadily increased over the three years [23]. Heavy metal toxicity significantly affects the growth and development of Chinese cabbage plants. The modifier can repair and improve the heavy metal soil to a certain extent, but with the increase in biochar addition, the growth of plants will decrease after a peak. The accumulation of polycyclic aromatic hydrocarbons will cause a certain degree of soil pollution, so plant germination and growth will not continue to increase with the increased addition of biochar [24].

### 4.2. Effects of Amendments on Soil Heavy Metal Concentration Changes

As soil amendments, biochar and organic fertilizer affect soil moisture according to their unique characteristics, such as pore structure, specific surface area, and soil texture. Generally, metal cations (Ca^2+^, Mg^2+^, K^+^, Na^+^) are electrostatically adsorbed on the biochar’s negatively charged active sites, complexing or precipitating with oxygen-containing functional groups on the surface. Heavy metals Cd^2+^ and Cu^2+^ in the soil may be adsorbed on the surface of biochar by ion exchange [25].

Soil water-holding capacity increases with the addition of soil amendments [26]. Eastman found that the bulk density of soils with biochar was significantly reduced [27]. With an increase in biochar and organic fertilizer, the soil bulk density and water permeability gradually decreased, and saturated water content gradually increased. Therefore, the heavy metal filtrate was not obtained from the BO 5% and BO 7% groups under normal irrigation conditions, which can be attributed to the enhanced water retention and reduced permeability resulting from the higher biochar content. However, a rainstorm occurred on day 21, leading to a notable increase in the concentration of heavy metals in the filtrate collected thereafter. This phenomenon can be explained by the following mechanisms: Firstly, intense rainfall can cause physical disruption of soil aggregates and promote the dispersion of clay particles, thereby increasing the mobility of heavy metals. Secondly, although biochar generally enhances the adsorption capacity of soil for heavy metals, under extreme rainfall conditions, the leaching effect may exceed the adsorption capacity of biochar, especially when the biochar’s adsorption sites approach saturation or when the flow rate prevents sufficient contact time for adsorption. Additionally, the temporary increase in heavy metal concentration may also be related to the re-release of previously adsorbed metals due to changes in soil solution chemistry and redox conditions induced by the rainstorm. The key explanation for this may be the local temperature fluctuation and continuous rainstorms that affected the adsorption of biochar [28]. In general, during a rainstorm, clay on the soil surface is dispersed, which further decomposes soil aggregates, resulting in an increase in the number of pores in the soil and significantly increasing the concentration of heavy metals in the leachate [29]. It was demonstrated in Jiang’s research that rainfall accelerates the leaching process of heavy metals [30]. Previous studies have shown that the addition of biochar can effectively enhance the passivation ability of soil against heavy metals. However, high-intensity rainfall can lead to serious soil erosion and the loss of heavy metals from the soil [31].

At the end of the rainstorm, the release of heavy metals in the soil returned to the pre-rainstorm level; however, the release of heavy metals in the CK and BO 0% groups without biochar remained high, primarily because rainfall causes the disintegration of soil aggregates. After the soil adsorption capacity reached saturation, large amounts of heavy metals were released. Therefore, in the BO 0% group, the presence of microbial organic fertilizer can effectively adsorb heavy metals.

With the combined addition of biochar and microbial organic fertilizer, the pH value of the soil increased to varying degrees. The experiments showed that the addition of biochar and microbial organic fertilizer may effectively regulate the pH of acidic soil. Biochar’s ability to increase soil pH is determined by the alkalinity of the biochar and by the organic acid root (-COO-) used in its manufacture [32].

### 4.3. Effects of Amendments on the Heavy Metal Speciation in Soil

The analysis of heavy metal forms showed that heavy metals are primarily in the residual fraction, which is basically consistent with other reports [33]. The exchangeable and carbonate-bound fraction forms of heavy metal cadmium accounted for a certain proportion, and the remaining forms were less than 5% or undetectable. This experiment revealed that the addition of biochar lowered the proportion of exchangeable fraction heavy metal Cd while marginally increasing the carbonate-bound fraction. The exchangeable and the carbonate-bound fractions in the soil decrease with increasing pH. The heavy metal Cu was primarily present in the residual and organic-bound fractions. The Fe-Mn oxide-bound fraction accounts for a relatively small proportion, and the remaining forms are less than 5% or undetectable. The addition of biochar significantly increases the proportion of organic-bound fraction of Cu ions, and the Fe-Mn oxide-bound fraction also increases with the increase in soil pH. Previous studies have shown that the passivation of heavy metals in soil is primarily affected by pH value. Alkaline soils reduce the availability of heavy metals (i.e., Cd, Cu, Ni, Pb, and Zn), thereby reducing the bioavailability and ecological toxicity of heavy metals in the soil [34].

### 4.4. Effects of Amendments on Soil Microbial Community Structure

Additionally, biochar provides additional nutrients and indirectly affects the microbial community by adsorbing nutrients or improving soil physical and chemical properties, thereby enriching the beneficial microbial populations in the soil. Microbial populations can produce a large number of antibiotics with antagonistic and inhibitory effects against pathogens [35]. The dominant bacterial genera in the soil were Proteobacteria, Acidobacteria, and Bacteroidetes.

Some laboratory and field studies have revealed the positive effects of biochar on microbial nitrogen fixation in soils [36]. However, the abundance of Proteobacteria, which mainly play a role in nitrogen fixation, decreased (5–40%) significantly in the pot experiments compared with that of the initial soil. This reduction in Proteobacteria in the pot experiment could be owing to the adequate soil moisture, which is known to decrease when the soil moisture content is high [37]. However, compared with the CK group, the abundance of Proteobacteria significantly increased, and biochar adsorption changed the availability of inorganic nitrogen. More importantly, it alters the microbially mediated nitrogen cycle. As the microbial community plays a role in nitrogen fixation, further exploration of the mechanisms of biochar-mediated Proteobacteria in the soil nitrogen cycle is necessary [38].

The abundance of Acidobacteria increased to approximately 15–32%, which was confirmed to be negatively correlated with soil Ph [39]. Studies on Acidobacteria are limited. Acidobacteria in soil are primarily associated with the degradation of plant residue polymers, the iron cycle, photosynthetic capacity, and metabolism of single carbon compounds. Our observation that biochar increased Proteobacteria abundance relative to the control is consistent with Tian et al. [40], who reported that biochar application influenced soil nitrogen transformation and microbial functional genes, including those associated with nitrogen-fixing bacteria.

The abundance of Bacteroidetes remained unchanged, whereas the abundances of Chloroflexi and Verrucomicrobia increased slightly. Microbial organic fertilizer can improve the soil microbial environment to a certain extent by adding exogenous microbial flora, so that the soil environment can be adjusted in a direction conducive to crop growth. According to research, Chloroflexi is more inclined to survive in a nutrient-rich soil environment. Chloroflexi are photoautotrophic bacteria that fix CO_2_ and generate energy to survive in various environments [41]. However, it is worth noting that Chloroflexi were more abundant in areas with high eutrophication [42]. Therefore, the increase in the abundance of Chloroflexi in the experimental group with amendments compared to the CK group indicates that the soil environment has been further improved. The addition of soil amendments significantly increased the abundance of Verrucomicrobia in topsoil. As a less studied microbial community, Verrucomicrobia has been shown to move downward with the soil profile. Verrucomicrobia play a key role in plant roots, the soil environmental carbon cycle, and polysaccharide degradation [43].

Studies have shown that microbial biomass decreases with increasing heavy metal content in soil. Patel study found that when the concentration of heavy metals reaches three times the standard concentration, they have a significant inhibitory effect on microorganisms [44]. The addition of soil amendments significantly optimized the abundance of dominant microbial communities in the soil. Studies have shown that the microorganisms in plant roots may be hotspots for DNA exchange. The addition of soil amendments regulated soil pH. Different types of microbial communities resist root acidification through pH regulation and provide carbohydrates. The results of pot experiments have revealed to a certain extent that the modifier can optimize the microbial abundance, improve the microbial environment of the soil, and reduce the inhibitory effects of heavy metals on microorganisms while remediating heavy metal pollution.

The addition of soil amendments strengthened the correlation between microbial community and biochar addition. It can be conjectured that biochar may play a key role in the symbiosis of exogenous microbial flora and soil microbial community. In future studies, it will be necessary to explore the transformation of microbial community symbiosis models using metagenomics.

## 5. Conclusions

This study focused on addressing three core objectives: identifying the optimal application ratio of biochar and microbial organic fertilizer, clarifying their synergistic mechanism in remediating heavy metal-contaminated soil, and verifying the applicability of this combined amendment. Guided by these objectives and targeting the research gap—an insufficient understanding of how different proportions of biochar and microbial organic fertilizer affect heavy metal adsorption and soil microbial abundance—the following key conclusions are drawn:

1. Optimal ratio confirmation: Under the experimental conditions (200 mg/kg Cu and 2 mg/kg Cd contamination), the combination of 3% biochar (soil weight basis) and 10% microbial organic fertilizer (soil weight basis) is the optimal ratio. This combination achieved the highest germination rate (34.78%) and plant height (12 cm) of Chinese cabbage, with Cu and Cd removal rates reaching 73.2% and 80%, respectively. Excessive biochar (5% and 7%) led to reduced plant growth, confirming that crop growth responses to biochar addition follow a “promotion then inhibition” pattern.
2. Synergistic remediation mechanism clarification: The combined amendment regulates soil pH from acidic (initial pH 5.4) to slightly alkaline (pH 7.2–7.5), reducing the exchangeable fraction of heavy metals (Cd exchangeable fraction decreased by ~10% compared to non-biochar groups) and increasing the residual fraction (Cu residual fraction accounting for 60–70%, Cd for 45–60%), thereby lowering heavy metal bioavailability and ecological toxicity. Biochar’s porous structure (observed via SEM) reduces soil compaction and provides habitats for microorganisms, while microbial organic fertilizer enriches exogenous beneficial flora. Together, they optimize soil microbial abundance: Acidobacteria (associated with plant residue degradation) increased to 15–32%, Chloroflexi (adapting to nutrient-rich environments) and Verrucomicrobia (involved in carbon cycling) showed enhanced abundance, and Proteobacteria (key for nitrogen fixation) was significantly higher than the control group, improving soil self-remediation capacity. The amendment enhances soil water-holding capacity and reduces bulk density, minimizing heavy metal leaching—particularly evident after rainfall, where biochar-amended groups showed 30–50% lower heavy metal concentrations in leachate compared to non-amended groups.
3. Applicability verification: The combined application of biochar and microbial organic fertilizer effectively addresses concurrent issues of heavy metal pollution and soil compaction in farmland. It creates a virtuous cycle: biochar adsorbs heavy metals and improves soil structure, microbial organic fertilizer regulates flora and enhances nutrient supply, collectively promoting crop growth and reducing heavy metal accumulation in plants (no detectable Cu/Cd in Chinese cabbage). This approach provides a practical and sustainable technical solution for the remediation of Cu/Cd-contaminated agricultural soils.

In summary, this study fills the research gap regarding the interactive effects of biochar-microbial organic fertilizer proportions on heavy metal adsorption and microbial communities. It confirms the optimal ratio, clarifies the synergistic remediation mechanism, and verifies field applicability, laying a theoretical and technical foundation for the sustainable management of heavy metal-contaminated farmland.

## Figures and Tables

**Table 1:** Physicochemical properties of microbial organic fertilizer.

Serial Number	Inspection Test Items	Unit	Inspection Test Results
1	Appearance		The color is brown, powdery. Uniform, odorless, inorganic Mechanical impurities.
2	Mass fraction of organic matter (dry basis), %		46
3	Total nutrient (nitrogen + phosphorus pentoxide + potassium oxide) mass fraction (based on drying), %		5.6
4	Moisture (fresh sample) mass fraction, %		27
5	pH		7.2
6	As (Measured by drying base)	mg/kg	0.1
7	Cu (Measured by drying base)	mg/kg	Not detected (detection limit: 0.01 mg/kg).
8	Pb (Measured by drying base)	mg/kg	2
9	Cd (Measured by drying base)	mg/kg	Not detected (detection limit: 0.01 mg/kg).
10	Cr (Measured by drying base)	mg/kg	15

## Data Availability

The data of this study are available upon request from the corresponding author.
